# Direct effects of prolonged TNF-α and IL-6 exposure on neural activity in human iPSC-derived neuron-astrocyte co-cultures

**DOI:** 10.3389/fncel.2025.1512591

**Published:** 2025-02-12

**Authors:** Noah Goshi, Doris Lam, Chandrakumar Bogguri, Vivek Kurien George, Aimy Sebastian, Jose Cadena, Nicole F. Leon, Nicholas R. Hum, Dina R. Weilhammer, Nicholas O. Fischer, Heather A. Enright

**Affiliations:** ^1^Physical and Life Sciences Directorate, Lawrence Livermore National Laboratory, Livermore, CA, United States; ^2^Engineering Directorate, Lawrence Livermore National Laboratory, Livermore, CA, United States

**Keywords:** multi-electrode array, neuron networks, human iPSC derived neurons, inflammation, cytokine, long-COVID, TNF-α, IL-6

## Abstract

Cognitive impairment is one of the many symptoms reported by individuals suffering from long-COVID and other post-viral infection disorders such as myalgic encephalomyelitis/chronic fatigue syndrome (ME/CFS). A common factor among these conditions is a sustained immune response and increased levels of inflammatory cytokines. Tumor necrosis factor alpha (TNF-α) and interleukin-6 (IL-6) are two such cytokines that are elevated in patients diagnosed with long-COVID and ME/CFS. In this study, we characterized the changes in neural functionality, secreted cytokine profiles, and gene expression in co-cultures of human iPSC-derived neurons and primary astrocytes in response to prolonged exposure to TNF-α and IL-6. We found that exposure to TNF-α produced both a concentration-independent and concentration-dependent response in neural activity. Burst duration was significantly reduced within a few days of exposure regardless of concentration (1 pg/mL – 100 ng/mL) but returned to baseline after 7 days. Treatment with low concentrations of TNF-α (e.g., 1 and 25 pg/mL) did not lead to changes in the secreted cytokine profile or gene expression but still resulted in significant changes to electrophysiological features such as interspike interval and burst duration. Conversely, treatment with high concentrations of TNF-α (e.g., 10 and 100 ng/mL) led to reduced spiking activity, which may be correlated to changes in neural health, gene expression, and increases in inflammatory cytokine secretion (e.g., IL-1β, IL-4, and CXCL-10) that were observed at higher TNF-α concentrations. Prolonged exposure to IL-6 led to changes in bursting features, with significant reduction in the number of spikes in bursts across a wide range of treatment concentrations (i.e., 1 pg/mL–10 ng/mL). In combination, the addition of IL-6 appears to counteract the changes to neural function induced by low concentrations of TNF-α, while at high concentrations of TNF-α the addition of IL-6 had little to no effect. Conversely, the changes to electrophysiological features induced by IL-6 were lost when the cultures were co-stimulated with TNF-α regardless of the concentration, suggesting that TNF-α may play a more pronounced role in altering neural function. These results indicate that increased concentrations of key inflammatory cytokines associated with long-COVID can directly impact neural function and may be a component of the cognitive impairment associated with long-COVID and other post-viral infection disorders.

## 1 Introduction

Following recovery from acute SARS-CoV-2 infection, many patients report lingering symptoms including fatigue, upper respiratory issues, gastroenterological symptoms, myalgia, insomnia, and neurocognitive issues (Raveendran, 2022). This is now recognized as ‘long-COVID’ or post-acute sequelae of SARS-CoV-2 infection (PASC) and is conservatively estimated to affect over 10% of COVID-19 patients (Ballering et al., 2022). Neurological symptoms comprise one aspect of long-COVID and manifest as trouble focusing and maintaining concentration, headaches, fatigue, forgetfulness, sensory disturbances, depression, and even psychosis (Kavanagh, 2022; Spudich and Nath, 2022). Many of the neurological symptoms of long-COVID closely resemble symptoms associated with other post-viral infection disorders such as myalgic encephalomyelitis/chronic fatigue syndrome (ME/CFS) (Stefano, 2021; Hirschenberger et al., 2021; Davis et al., 2023; Wong and Weitzer, 2021) or symptoms described by patients who have cancer-therapy-related cognitive impairment for non-central nervous system related cancer, termed ‘chemo fog’ (Wefel et al., 2015; Fernández-Castañeda et al., 2022). While there is significant debate on whether this sustained immune response is the primary mechanism responsible for long-COVID or a byproduct of other underlying mechanisms (Altmann et al., 2023), a common theme among these disorders appears to be sustained inflammatory cascades that persist beyond the resolution of the initial insult (Clark, 2022). Furthermore, these long-lasting alterations to systemic immunity and cytokine and chemokine profiles have been shown to have direct neurotoxic effects, influence microglia activity, and alter neuron activity and connectivity (Seigers et al., 2013; Fernández-Castañeda et al., 2022; Wefel et al., 2015; Murillo et al., 2023). Collectively, this suggests the potential for a common pathophysiological mechanism responsible for the neurological symptoms present in the aforementioned disorders.

Tumor necrosis factor alpha (TNF-α) and interleukin-6 (IL-6) are proinflammatory cytokines that are elevated in patients with long-COVID (Schultheiss et al., 2022; Fernández-Castañeda et al., 2022), ‘chemo fog’ (Ganz et al., 2013; Kesler et al., 2013; Pomykala et al., 2013; Murillo et al., 2023), and other post-viral infection disorders (Islam et al., 2020; Garcia et al., 2014; Mowbray and Yousef, 1991). TNF-α and IL-6 have also been investigated as biomarkers for long-COVID, especially in relationship to long-lasting neurocognitive symptoms (Comeau et al., 2023). Serum levels of TNF-α and IL-6 were significantly elevated in patients self-reporting neurocognitive symptoms lasting >90 days after infection (Peluso et al., 2022). Similarly, patients with ongoing long-COVID at 8 months showed significantly higher plasma levels of TNF-α and IL-6 as compared to those who were never infected or never showed symptoms of long-COVID (Schultheiss et al., 2022). Both cytokines are pleiotropic, contributing to normal physiological conditions in the brain at low concentrations but are found to be upregulated in brain diseases and injuries (Khaboushan et al., 2022; Hong et al., 2016; Becher et al., 2017). In addition to their role as immune-associated cytokines, both TNF-α and IL-6 have been shown to alter ion channel expression, interact directly with neuron ion channels, and modulate neural activity in the absence of immune cells (Vezzani and Viviani, 2015; Marin and Kipnis, 2013). The goal of this study is to determine if elevated TNF-α and IL-6 levels associated with long-COVID can affect neural activity and modulate the functional dynamics of neural network connectivity in co-cultures of human iPSC-derived neurons and primary astrocytes, which may provide insight into the neurocognitive effects of long-COVID.

In this study, we seeded co-cultures of human iPSC-derived neurons and primary astrocytes with multielectrode array (MEA) technology to evaluate how sustained exposure to TNF-α and/or IL-6 modulates neuronal network activity. This system is capable of non-invasively monitoring fast neuronal dynamics at single cell, network (i.e., the communication between neurons), and community levels (i.e., communication within a cluster of networks) with high spatiotemporal precision for a prolonged period of time (Anderson et al., 2021; Bang et al., 2019). Previous studies have used similar systems to examine the short-term consequences of TNF-α (<24 h) and IL-6 exposure (<48 h) on neural activity (Gullo et al., 2014; Jewett et al., 2015; Clarkson et al., 2017; Dykstra-Aiello et al., 2021; Black et al., 2018); however, the effect of prolonged exposure to these cytokines at physiologically relevant concentrations has yet to be determined. In addition to investigating neuronal activity, we also compared transcriptomic and cytokine/chemokine response of the cultures to TNF-α and IL-6 exposure. We found that exposure to TNF-α produced both a concentration-independent and concentration-dependent response in neural activity. Burst duration was significantly reduced within a few days of exposure regardless of concentration but returned to baseline after 7 days. Conversely, spiking activity decreased at later timepoints but only at higher concentrations, which may be related to changes in neuronal health along with changes to the transcriptional and cytokine/chemokine profile that was only observed at higher TNF-α concentrations. For IL-6, we saw a significant decrease in the total number of spikes in bursts across all concentrations and timepoints. Interestingly, when cultures were co-stimulated with both TNF-α and IL-6, at low TNF-α concentrations, the addition of IL-6 appears to counteract the changes to neural function induced by low concentrations of TNF-α, while at high concentrations of TNF-α the addition of IL-6 had little to no effect. Conversely, the changes to electrophysiological features induced by IL-6 were lost when the cultures were co-stimulated with TNF-α regardless of concentration, suggesting that TNF-α may have a larger impact on neuron function especially at higher concentrations.

## 2 Methods

### 2.1 Cell culture

The cell culture system used in this study followed vendor (NeuCyte Inc.) recommended protocols for their standard SynFire co-culture platform. In short, human iPSC-derived glutamatergic and GABAergic neurons (at a ratio of 70:30) were co-cultured with primary human astrocytes at a ratio of 3:1 neurons:astrocytes (NeuCyte Inc.), as in previous studies (Bogguri et al., 2024; Lam et al., 2023; Soscia et al., 2020). The total seeding density was 3,125 cells/mm^2^ based on vendor recommendation and previous studies conducted by our group (Bogguri et al., 2024) and others (Sasaki et al., 2019; Wang et al., 2023). Briefly, 6-well layout MEA devices (MEA200/30iR-ITO, Multi-channel Systems) were plasma-treated (PDC-001-HP, Harrick Plasma) for 3 min before being soaked in phosphate buffered saline with calcium and magnesium (PBS+; Sigma Aldrich) overnight at 30°C. The MEAs were washed with sterile DI water (4X) and then air dried before autoclaving at 121°C. Both MEA devices and 96 flat bottom-well plates were coated with 0.1% PEI (prepared in borate buffer) for overnight incubation at 37°C, washed with sterile DI water (4X), then coated with a 20 μg/mL of laminin solution in PBS without calcium and magnesium for 2 h at 37°C. The laminin solution was removed before cells were seeded. Purified and concentrated stocks of neuronal subtypes and astrocytes were thawed and diluted, and live cell counts were obtained using a Countess Automated Cell Counter. The appropriate volumes of live cell populations for each cell type were pooled to achieve cell type specific ratios, spun down at 250 g for 5 min, and resuspended in seeding media (NeuCyte Inc.) before deposition in the wells of the MEA device or 96-flat bottom-well plate (50 μL per well). Cultures were maintained in a humidified incubator (37°C, 5% CO_2_). After 24 h, 150 μL of short-term media (NeuCyte Inc.) was added to each well, after which 50% of media was replaced every 2–3 days for culture maintenance. After 1 week in culture, media changes were performed using long-term media (NeuCyte Inc.) for the duration of the experiment. All media was used as supplied by the vendor with no alterations.

### 2.2 Cytokine exposure

Recombinant human TNF-α (R&D systems) and IL-6 (R&D systems) were reconstituted to a stock concentration of 100 μg/mL using PBS with 0.1% w/v bovine serum albumin (BSA) and were stored at −20°C until use for experiments. At ~25 days *in vitro* (DIV), a 50% media exchange was conducted, adding TNF-α, IL-6, or both. A 2X working stock solution was prepared in culture media before added to the cultures for the final TNF-α (1 pg/mL, 25 pg/mL, 100 pg/mL, 10 ng/mL, or 100 ng/mL) or IL-6 (1 pg/mL, 10 pg/mL, 10 ng/mL, 50 ng/mL, or 100 ng/mL) concentration used in the study. All controls had fresh neuron media with an equivalent volume of 0.1% BSA in PBS to act as the vehicle control and to mimic the mechanical perturbations that result from dosing the cultures with the cytokines. Two days after the initial cytokine exposure, another 50% media exchange was conducted once more with the same 2X concentration of cytokine or media added for the respective treatment conditions. Cultures were exposed to cytokine(s) for a period of 7 days.

### 2.3 Multi-electrode array recordings

For recordings, the 6-well MEA device was placed within a 5% CO_2_-regulated chamber on the heated stage (37°C) of a 256-channel MEA2100 recording system (Multichannel Systems). Following a 5-min equilibration time, electrophysiology activity was recorded for 30 min at a sampling frequency of 10 kHz and bandpass filtered between 4 and 4,000 Hz, as before by our group (Lam et al., 2019; Enright et al., 2020; Soscia et al., 2020; Soscia et al., 2017) and others (Novellino et al., 2011; Pastore et al., 2018). An action potential spike was defined by a lower limit threshold, set at 6.5x the standard deviation of baseline noise, for each electrode. Devices were recorded for 30 min once a week from 7 DIV and onwards to monitor the development and maturation of neural networks. Cytokine exposure experiments were conducted at ~25 DIV, a time point in which activity becomes stable (Enright et al., 2020; Lam et al., 2019). At this time point, baseline activity of the 6-well MEA device was recorded and then wells are randomly assigned for TNF-α, IL-6, or co-stimulation with TNF-α and IL-6. Following treatment with the cytokine(s), recordings were conducted within the first hour of exposure (i.e., 2 × 30 min recordings), and at 24 h, 48 h, and 7 days post-exposure.

### 2.4 Feature analysis

As is previous studies (Enright et al., 2020; Lam et al., 2019; Soscia et al., 2017; Soscia et al., 2020), time-stamped data from each recording was exported as a HDF5 file and analyzed using an in-house custom R package. Burst detection parameters were defined based on previous publications by Charlesworth et al. (2015) and Chiappalone et al. (2005). Electrodes were included in the analysis if they recorded at least 10 spikes in the 30 min recording window. The burst parameters included: maximum beginning interspike interval (ISI) of 0.1 s, maximum end ISI of 0.2 s, minimum interburst interval (IBI) of 0.5 s, minimum burst duration of 0.05 s, and minimum number of spikes per burst of 6. Additional parameters included the removal of any electrodes with a mean burst duration greater than 5 s to eliminate potentially noisy electrodes (Bogguri et al., 2024). For electrodes within an array of a well that had no detectable spiking or bursting activity, a value of ‘0’ was determined. To minimize the effect of mechanical disturbance, attributed to pipetting in the cytokine solution, the mean (for a specific feature) prior to cytokine exposure (e.g., baseline) was calculated. Then, the mean following cytokine exposure was calculated and normalized to baseline activity. The values for the cytokine-treated wells (normalized to baseline) are expressed as a fold change relative to the average value from the 0 pg/mL cytokine concentration (normalized to baseline) at the same time point (e.g., age-matched) and used for further statistical analysis.

Coordinated activity between a pair of electrodes was calculated using SPIKE-distance as previously described (Kreuz et al., 2013). SPIKE-distance measures the dissimilarity between two spike trains as the average of the *instantaneous* dissimilarity between the two spike trains at different points of the recording. As in previous studies (Eisenman et al., 2015; Enright et al., 2020; Lam et al., 2019), spike train distances were subtracted from 1 to obtain a similarity or synchrony measure, such that a value of 1 represents perfect synchrony and a value of 0 denotes complete asynchrony. Additionally, values were normalized by the SPIKE-distance obtained on randomly generated spike trains to compensate for the documented bias of SPIKE distance to assign higher synchrony values to denser spike trains (Sihn and Kim, 2019). As before with spiking and bursting features, electrodes with a mean burst duration greater than 5s were eliminated to remove noisy electrodes from the SPIKE distance analysis. Less than 1% of electrodes across all experiments were removed by this criterion.

### 2.5 Lactate dehydrogenase (LDH) assay

CyQuant™ Lactate Dehydrogenase (LDH) assay (Thermo Fisher Scientific) was performed according to the manufacturer’s instructions. Fresh culture supernatant was collected at 2 and 7 DIV and LDH activity was measured spectrophotometrically at 490 and 680 nm on the Synergy H1 multi-mode microplate reader (BioTek). The absorbance data for cytokine treatment was normalized to the untreated condition.

### 2.6 Multiplex cytokine and chemokine ELISA

Cytokines were quantified using LEGENDplex™ Human Essential Immune Response panel, a multiplex fluorescence-encoded bead-based assay (BioLegend). Samples were prepared according to manufacturer’s instructions. Flow cytometry of the beads was performed using a FACS Aria Fusion (BD Biosciences), and data analyzed using BioLegend’s cloud-based analysis software^1^.

### 2.7 Bulk RNA-sequencing

Cultures were exposed to 25 pg/mL, 10 ng/mL, 100 ng/mL of TNF-α, or vehicle for 7 days before lysed using RLT buffer containing β-mercaptoethanol and RNA was isolated using the RNAeasy mini spin columns (Qiagen). Illumina Stranded mRNA Prep kit (Illumina) was used to prepare the sequencing libraries and sequencing was performed using an Illumina NextSeq 2000. The quality of sequencing data was checked using FastQC software^2^. The reads were mapped to the human genome (hg38) using STAR and read counts per gene were determined using “featureCounts” from Rsubread package (Liao et al., 2014; Risso et al., 2014). Subsequently, differentially expressed genes were identified using the limma package with its voom method (Law et al., 2016; Ritchie et al., 2015). A gene was considered as significantly differentially expressed when its false discovery rate adjusted *p*-value was <0.05 and fold change was >1.5. Gene ontology (GO) analysis was performed using ToppGene (Chen et al., 2009). Heatmaps were generated using heatmap.2 function in “gplots” R package. Volcano plots were generated using Galaxy Europe^3^.

### 2.8 Statistical analysis

Data is expressed as mean ± standard error of the mean (SEM) for the number of replicates indicated, unless stated differently. Electrophysiological features were determined on a per-electrode basis (or electrode pair basis for synchrony), but statistical analysis was performed on a per-well basis by taking the overall mean from the active electrodes. For electrophysiology, LDH, and cytokine/chemokine experiments statistical significance was analyzed in GraphPad version 8 (GraphPad Software) using a one-way ANOVA or a mixed model repeated measures two-way ANOVA with Dunnett’s or Tukey’s *post hoc* analysis. For all experiments, statistical significance was determined by *p*-values <0.05. For bulk RNA-sequencing datasets statistical significance was determined as described above.

## 3 Results

### 3.1 Functional characterization of neural networks following TNF-α exposure

Elevated levels of TNF-α, IL-8, and IL-6, have been detected in hospitalized patients during the initial stages of SARS-CoV-2 infection, and TNF-α and IL-6, in particular, are significant predictors for increased severity and death (Del Valle et al., 2020). Here, we conducted a concentration-response study to assess how human iPSC-derived neural networks are modulated by prolonged exposure to TNF-α at clinically relevant concentrations. We tested concentrations of 1 and 25 pg/mL, which was in range with control patients (Del Valle et al., 2020; Hirzel et al., 2022; Schultheiss et al., 2022), and 100 pg/mL, which is in range of hospitalized COVID patients and patients suffering from symptoms of long-COVID (Torabi et al., 2020; Jarius et al., 2022; Ramezani et al., 2022; Schultheiss et al., 2022). We also evaluated 10 ng/mL and 100 ng/mL TNF-α, concentrations reported from previous *in vitro* studies using human iPSC-derived or neuroblastoma derived cells (Talley et al., 1995; Hyvärinen et al., 2019; Jayaraman et al., 2021; Saraf et al., 2021; Kerkering et al., 2023). Neurons and astrocytes were seeded at ratios outlined in the methods to model the cortical region of the brain (Defelipe and Farinas, 1992; Markram et al., 2004). Bulk RNA sequencing data confirmed the detection of neuronal markers, tubulin beta 3 (TUBB3) and RNA binding fox-1 homolog 3 (RBFOX3), and the astrocyte marker, glial fibrillary acidic protein (GFAP), from the co-culture system as shown in Supplementary Figure S1.

**Figure 1:**
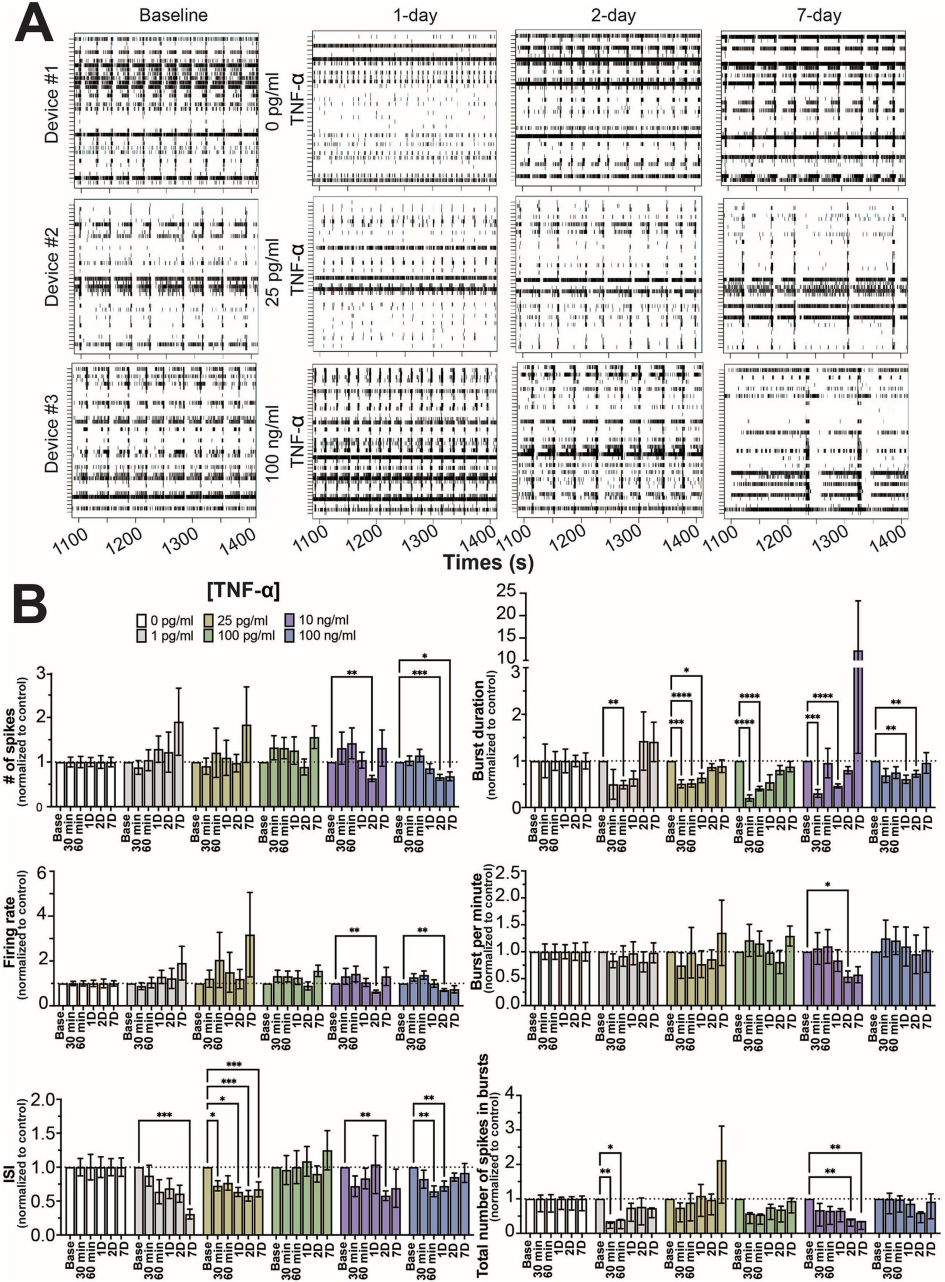
Concentration-dependent changes in neural activity during prolonged TNF-α exposure. **(A)** Representative five-minute raster plots from control, 25 pg/mL, and 100 ng/mL TNF-α treatments at 1, 2, and 7 days of exposure. Baseline recordings before cytokine exposure were taken at 25 days in culture. **(B)** Comparison of different spiking and bursting features across TNF-α treatment concentrations and exposure times (30 and 60 min), 1-(1D), 2-(2D), and 7 days (7D) of exposure (*n* = 6–18 wells/condition from at least 4 independent seedings). Data from each treatment condition were normalized to the age-matched untreated (control) conditions (dashed line) and shown as mean ± SEM; *p < 0.05, **p < 0.01, ***p < 0.001, and ****p < 0.0001 (as determined by a repeated measures two-way ANOVA followed by Dunnett’s test vs. baseline for each treatment concentration).

As in previous studies (Lam et al., 2019; Lam et al., 2022), the number of active electrodes became stable around 21 days in culture (Supplementary Figure S2) and mature neural and network activity, as indicated by coordinated spiking and bursting activity, is detected by day 25 (Figure 1A). Co-cultures on the MEA devices were selected at random and were treated with one of 6 concentrations of TNF-α (0 pg/mL, 1 pg/mL, 25 pg/mL, 100 pg/mL, 10 ng/mL, and 100 ng/mL). Thirty minute recordings were taken after 30 min, 60 min, 1, 2, and 7 days of exposure to TNF-α. Figure 1A displays representative raster plots of spiking and bursting patterns from three different TNF-α treatment concentrations (control, 25 pg/mL, and 100 ng/mL) across multiple days of exposure.

Mechanical disturbances caused by pipetting have been shown to affect neural network activity in culture (Lam et al., 2022). To minimize the effect of mechanical disturbance, time points for each feature were normalized to age-matched controls. When cultures were exposed to TNF-α at nanogram concentrations (e.g., 10 and 100 ng/mL), features of spiking activity (number of spikes, firing rate, and ISI) decreased at intermediate timepoints (24 and 48 h) but largely returned to control levels by 7 days of exposure (Figure 1B). Interestingly, at picogram concentrations that are within range of normal clinical levels (Del Valle et al., 2020; Hirzel et al., 2022; Schultheiss et al., 2022), ISI changes persisted throughout the length of the experiment (Figure 1B). For the 1 pg/mL condition, ISI trended down throughout the experiment, but did not become significantly decreased until day 7. For the 25 pg/mL condition, a significant decrease in ISI was observed 30 min post treatment, which remained throughout the 7 days of treatment (Figure 1B). No change in spiking activity was observed in the 100 pg/mL condition. For features of bursting activity (burst duration, bursts per minute, and total number of spikes in bursts) the presence of TNF-α, independent of concentration, decreased burst duration at early timepoints, which recovered by day 7 (Figure 1B). While we did observe a few combinations of timepoints and concentrations that showed statistically significant differences, overall the total number of spikes in bursts and bursts per minute were not affected by TNF-α treatment.

**Figure 2:**
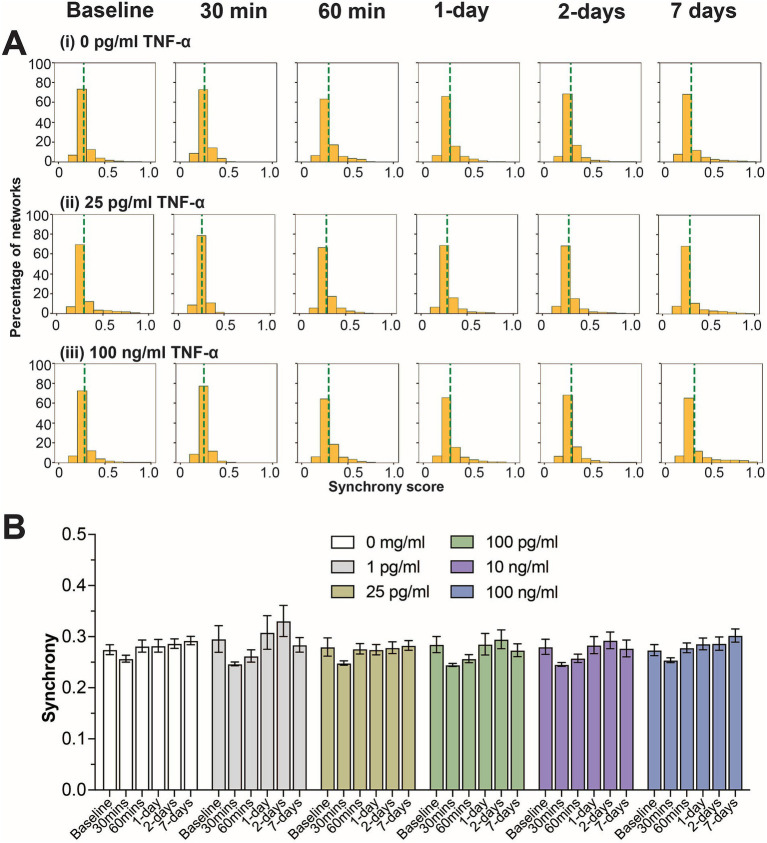
Influence of prolonged TNF-α exposure on network synchrony. **(A)** Histograms summarizing the distribution of synchrony scores across all electrode parings for control, 25 pg/mL, and 100 pg/mL TNF-α treatments. The dashed green line indicates the average synchrony score from all electrode pairs. **(B)** Comparing the influence of prolonged TNF-α exposure on average synchrony scores showing n.s. difference in average synchrony scores across any timepoint or concentration (as determined by a repeated measure two-way ANOVA). Data are shown as mean ± SEM.

Next, we examined how TNF-α treatment affected the degree of coordination (or synchrony) in spiking activity between networks within the co-culture system. As previously described (Cadena et al., 2020; Enright et al., 2020; Lam et al., 2019; Lam et al., 2022), the SPIKE distance method (Kreuz et al., 2013) was used to score all possible electrode parings for a given 30 min recording within each MEA. A score of ‘0’ indicates that the pair of electrodes has no synchrony, while a score closer to ‘1’ indicates a high degree of synchrony. We observed a consistent unimodal distribution of synchrony scores across all timepoints and TNF-α concentrations (Figure 2A and Supplementary Figure S3). Similarly, no significant difference was observed when comparing the average synchrony score, which ranges from 0.25 to 0.35, across each concentration and time points (Figure 2B).

### 3.2 Functional characterization of neural networks following IL-6 exposure

**Figure 3:**
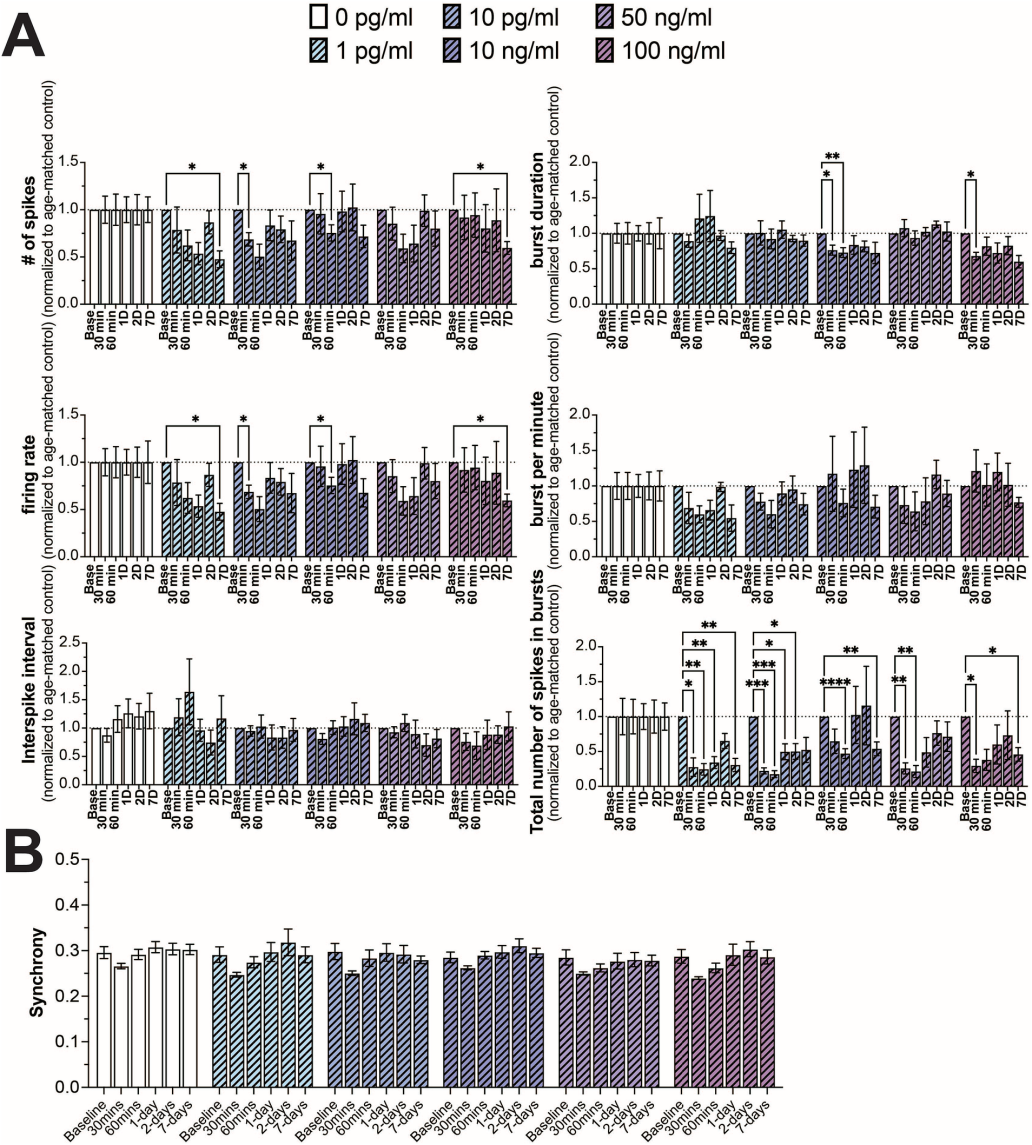
Comparing changes in neural activity during prolonged IL-6 exposure. **(A)** Comparison of different spiking and bursting features across IL-6 treatment concentrations and exposure times (*n* = 4–15 wells/condition from at least 3 independent seedings). Data from each treatment condition were normalized to the age-matched untreated (control) conditions (dashed line). **(B)** Comparing the influence of prolonged IL-6 exposure on average synchrony scores showing n.s. difference in average synchrony scores across any timepoint or concentration. Data are shown as mean ± SEM; *p < 0.05, **p < 0.01, ***p < 0.001, and ****p < 0.0001 (as determined by a repeated measures two-way ANOVA followed by Dunnett’s test vs. baseline for each treatment concentration).

In addition to TNF-α, we also investigated the effect of IL-6 on neuron functionality, comparing a similar range of concentrations spanning control (0 and 1 pg/mL) (Han et al., 2020), hospitalized COVID patients and patients suffering from long-COVID (10 pg/mL) (Han et al., 2020; Donoso-Navarro et al., 2021; Ulhaq and Soraya, 2020; Schultheiss et al., 2022), along with concentrations shown to affect neural activity in culture (10, 50, and 100 ng/mL) (Vereyken et al., 2007; Nelson et al., 2002; D’Arcangelo et al., 2000; Kawasaki et al., 2008). Interestingly, in contrast to TNF-α, we did not observe a strong concentration-dependent effect on any of the measured electrophysiological features. We observed a general reduction in neural activity (number of spikes and firing rate) across all concentrations of IL-6, however significant differences were only observed sporadically at varying timepoints and concentrations, suggesting a broad, but minimal reduction in neural activity in response to IL-6 (Figure 3A). We found a more robust response to IL-6 exposure on the total number of spikes in bursts, with a significant reduction in at least two timepoints across all concentrations (Figure 3A). Similar to TNF-α, we did not observe a significant change in the distribution of synchrony scores (Supplementary Figure S4) or average synchrony score (Figure 3B) at any concentration or exposure time.

### 3.3 Functional characterization of neural networks following co-stimulation of TNF-α and IL-6

As TNF-α and IL-6 are both elevated in patients with long-COVID (Fernández-Castañeda et al., 2022; Schultheiss et al., 2022), we wanted to determine if the combination of both TNF-α and IL-6 exposure may lead to more significant effects on network activity. As we did not see a strong concentration-dependent effect with IL-6 exposure, we exposed the cultures to a combination of 10 ng/mL IL-6 [the lowest tested concentration that has been previously shown to affect neuron activity in culture (Vereyken et al., 2007; Nelson et al., 2002; D’Arcangelo et al., 2000; Kawasaki et al., 2008)] and either 25 pg/mL or 100 ng/mL TNF-α. These two concentrations of TNF-α were chosen as cultures exposed to 25 pg/mL did not show a significant change in firing rate (concentration-dependent effect) but did show changes to ISI and burst duration (concentration-independent effects), while cultures exposed to 100 ng/mL showed changes to both firing rate and ISI and burst duration.

**Figure 4:**
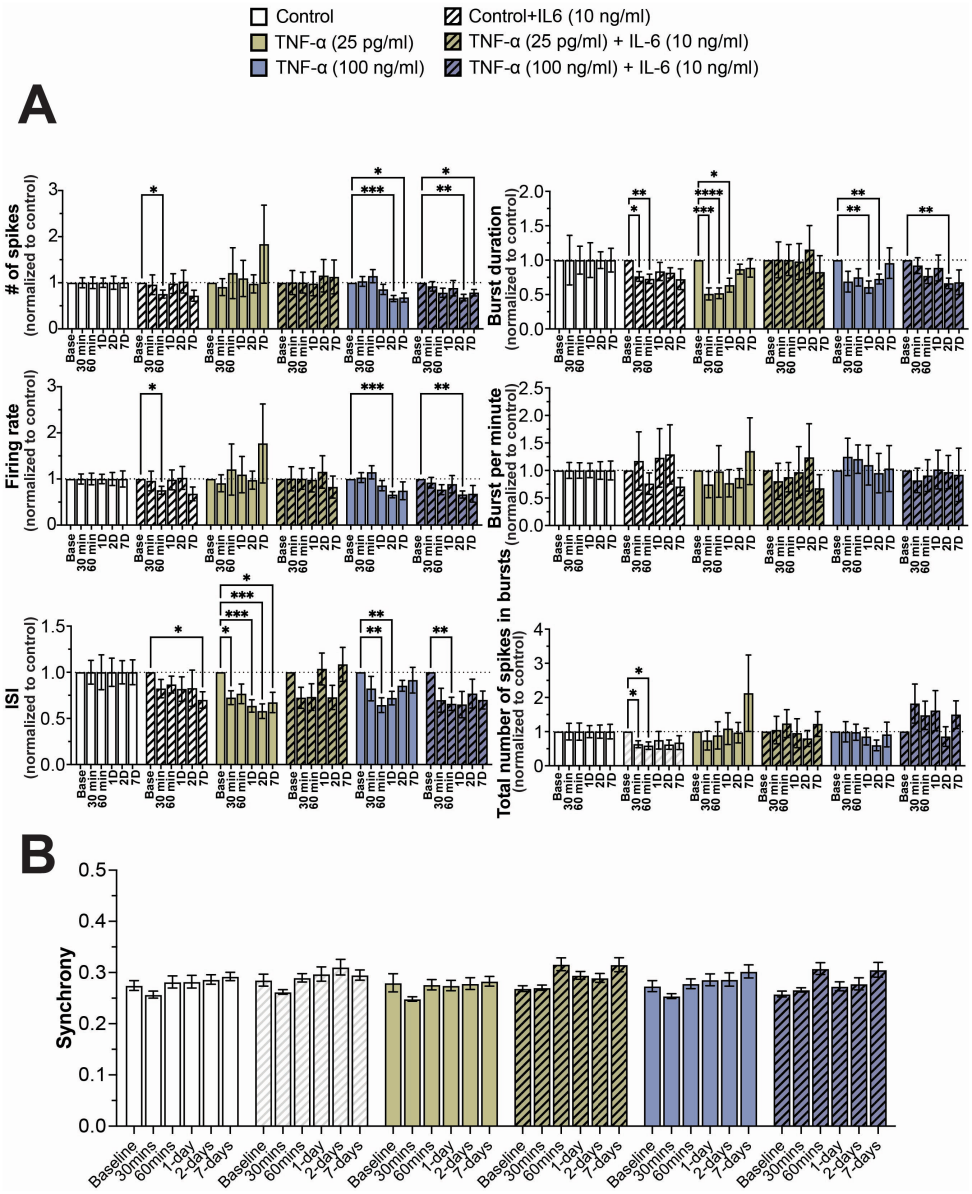
Concentration-dependent changes in neural activity during prolonged exposure to both TNF-α and IL-6. **(A)** Comparison of different spiking and bursting features across treatment concentrations and exposure times (*n* = 10–18 wells/condition from at least 3 independent seedings). Data from each treatment condition was normalized to the age-matched untreated (control) conditions (dashed line). **(B)** Comparing the influence of prolonged exposure on average synchrony scores showing n.s. difference in average synchrony scores across any timepoint or condition. Data are shown as mean ± SEM; *p < 0.05, **p < 0.01, ***p < 0.001, and ****p < 0.0001 (as determined by a repeated measures two-way ANOVA followed by Dunnett’s test vs. baseline for each treatment concentration).

As before, we observed a general reduction in neural activity and total number of spikes in bursts when cultures were exposed to 10 ng/mL IL-6, with significant decreases in these features at sporadic timepoints (Figure 4A). Similarly, we observed a significant decrease in ISI and burst duration in cultures exposed to both 25 pg/mL and 100 ng/mL TNF-α, but only detected a significant decrease in firing rate and total number of spikes in cultures exposed to 100 ng/mL at later timepoints (Figure 4A). When cultures were exposed to 25 pg/mL TNF-α in combination with 10 ng/mL IL-6, we observed no significant change in any electrophysiological feature at any timepoint, suggesting that TNF-α and IL-6 may have opposing effects on network activity (Figure 4A). However, when these systems were exposed to 100 ng/mL TNF-α with 10 ng/mL IL-6, the changes in electrophysiological features were nearly identical to that of just 100 ng/mL TNF-α, suggesting that TNF-α plays a dominant role at higher concentrations (Figure 4A). Finally, just as before, we observe no significant changes in the distribution of synchrony scores (Supplementary Figure S5) or average synchrony score (Figure 4B) at any exposure combination.

### 3.4 Cell viability was impacted at high concentrations of TNF-α, but not when combined with IL-6

**Figure 5:**
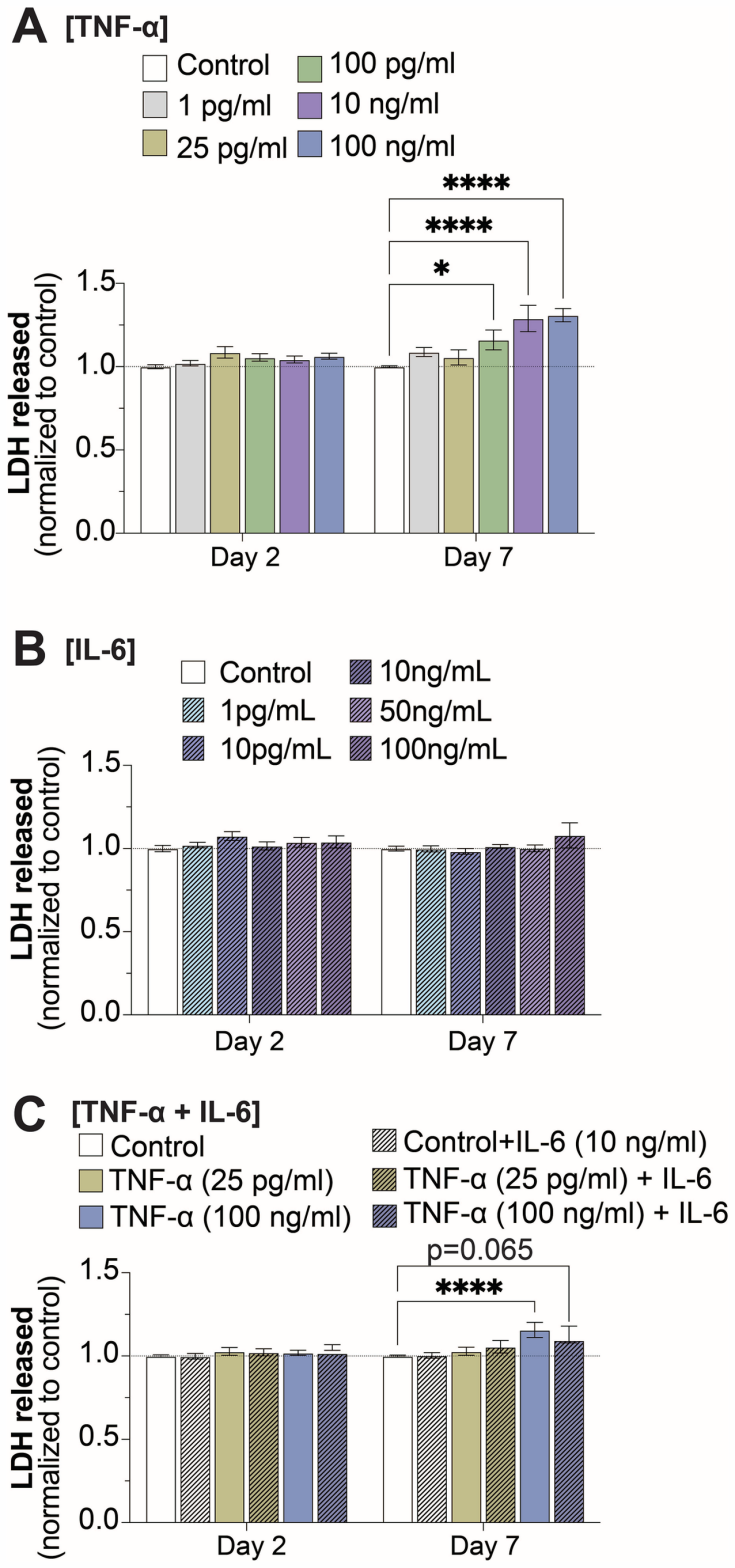
Changes in cell viability at 2- and 7-days of prolonged exposure **(A)** TNF-α, **(B)** IL-6, and **(C)** combined TNF-α and IL-6 exposure (*n* = 4–24 from at least 4 independent seedings). Data is normalized to age-matched control condition (dotted white line) and are shown as mean ± SEM; *p < 0.05, **p < 0.01, ***p < 0.001, and ****p < 0.0001 (as determined by a two-way ANOVA for each timepoint followed by Dunnett’s test vs. baseline for each treatment concentration).

In order to determine if the changes to electrophysiological features was a consequence of cell death, we quantified extracellular LDH at 2- and 7-days of exposure for all TNF-α and IL-6 concentrations. We observed a significant increase in extracellular LDH at 7-days of exposure to 100 pg/mL, 10 ng/mL, and 100 ng/mL TNF-α, but not for lower concentrations or at 2-days of exposure (Figures 5A,C). No change in cell viability was observed at either 2- or 7-days of exposure to any concentration of IL-6 (Figure 5B). Finally, co-stimulation with IL-6 somewhat reduced the cytotoxic effects of prolonged exposure to high concentrations of TNF-α, as cultures exposed to 100 ng/mL of TNF-α in combination with 10 ng/mL IL-6 no longer show a significant increase in extracellular LDH at day 7 as compared to untreated controls (Figure 5C).

### 3.5 Inflammatory cytokines

**Figure 6:**
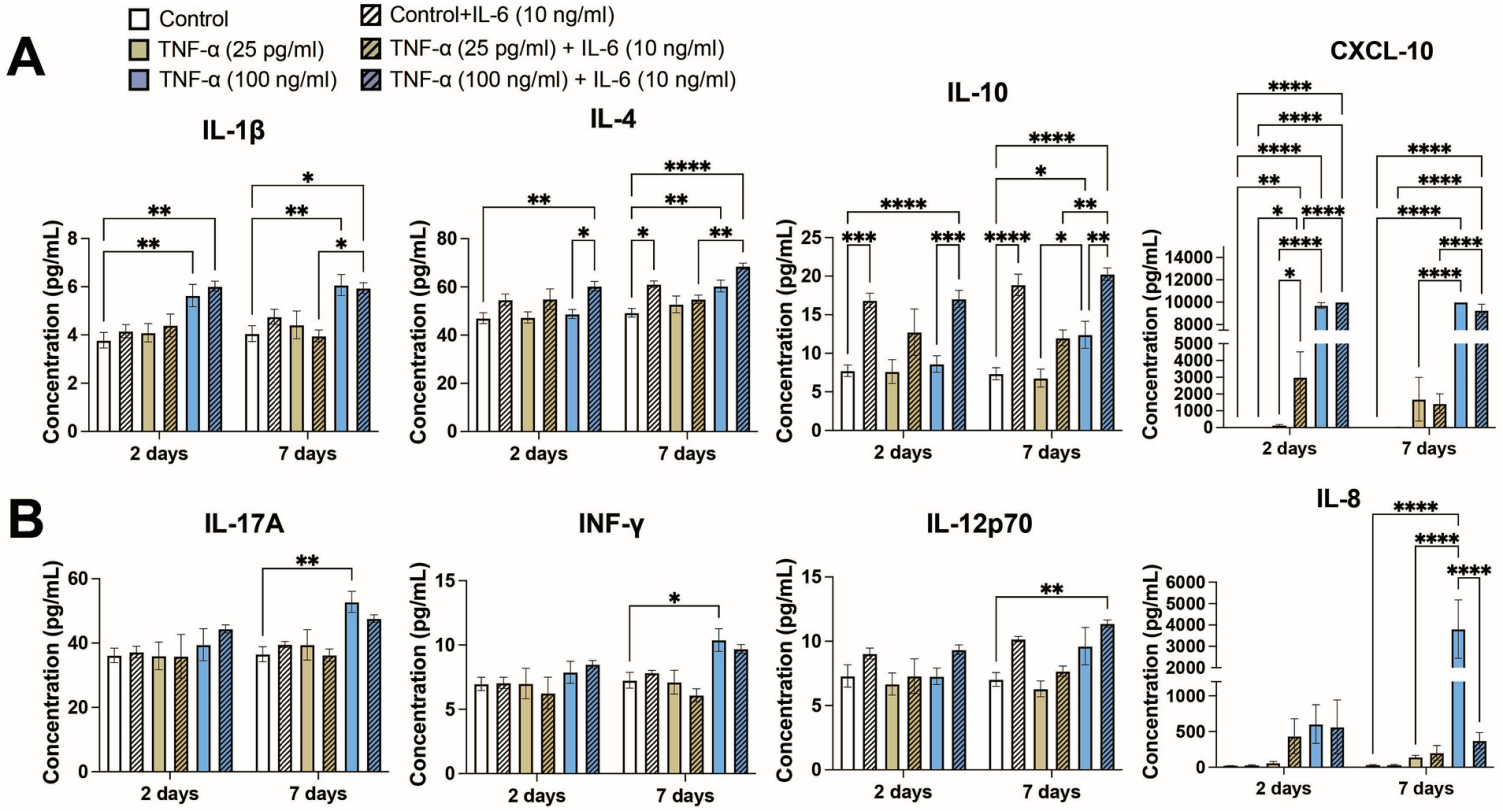
Concentrations of key cytokines associated with the immune response from conditioned media of co-cultures exposed to both TNF-α and IL-6 that show significant changes at **(A)** 48 h and **(B)** 7 days of exposure (*n* = 4–12 from at least 2 independent seedings). Data are shown as mean ± SEM; *p < 0.05, **p < 0.01, ***p < 0.001, and ****p < 0.0001 (as determined by a one-way ANOVA for each timepoint followed by a *post hoc* Tukey’s test). Some significant post hoc comparisons, in which the treatment conditions have no overlap (i.e., 25 pg/mL TNF-α + 10 ng/mL IL-6 vs. 100 ng/mL TNF-α), are not shown to improve clarity.

Due to the prolonged exposure times, we examined whether other cytokines secreted by the co-culture may have contributed to modulating neural activity. We compared cytokine secretion profiles of 13 key cytokines associated with the immune response (IL-4, IL-2, CXCL-10, IL-1β, IL-6, TNF-α, MCP-1, IL-17A, IL-10, IFN-γ, IL-12p70, TGF-β, and IL-8) following exposure to 25 pg/mL and 100 ng/mL TNF-α with and without co-stimulation with 10 ng/mL IL-6. At 2-days of exposure we found that 4 cytokines (IL-4, CXCL-10, IL-1β, and IL-10) showed significantly different secretion levels based on treatment condition (Figure 6A), while at 7-days of exposure we found that 8 cytokines had significantly different secretion levels (IL-4, IL-2, CXCL-10, IL-1β, IL-17A, IL-10, IFN-γ, IL-12p70, and IL-8; Figure 6). As expected, exposure conditions containing 100 ng/mL TNF-α and/or 10 ng/mL IL-6 had high concentrations of TNF-α and IL-6, respectively. Additionally, cultures exposed to 100 ng/mL TNF-α showed a significant increase in the concentration of IL-6 in the culture media, while cultures exposed to 10 ng/mL IL-6 did not show any change in TNF-α concentration (Supplementary Figure S5). Interestingly, we observed ~180 pg/mL of IL-6 in the conditioned media in the control conditions, while the average concentration of TNF-α was found to be at or less than the lower limit of detection for the assay (<2.5 pg/mL; Supplementary Figure S6).

Of the 4 cytokines that showed significantly different expression levels at 48 h (Figure 6A), increases in CXCL10 and IL-1β were primarily a response to treatment with 100 ng/mL TNF-α. Treatment with IL-6 significantly increased the levels of CXCL10 as compared to the corresponding untreated condition or 25 pg/mL TNF-α, however were unable to observe any potential further increase in CXCL10 concentration via co-stimulation of IL-6 with 100 ng/mL TNF-α as it was at or beyond the upper limit of detection. Co-stimulation with IL-6 did not change IL-1β concentration. Significant increases of IL-10 was primarily dependent on IL-6 treatment, as cultures treated with only TNF-α showed minimal changes in IL-10 concentration as compared to untreated control. Interestingly, increases in IL-4 seemed to be dependent on co-stimulation with both 100 ng/mL TNF-α and 10 ng/mL IL-6 as that conditioned showed not only a significant increase in IL-4 secretion as compared to the control, but also as compared to the 100 ng/mL TNF-α treatment condition. For each of these cytokines, the changes in cytokine levels at 7 days largely matched the changes seen at 48 h.

In addition to the four aforementioned cytokines, an additional four cytokines were significantly increased in the culture media following 7 days of TNF-α and IL-6 exposure (Figure 6B). Changes in IFN-γ and IL-17A showed similar patterns, with both cytokines showing significant increases in their concentration being primarily dependent on exposure to 100 ng/mL TNF-α. For both cytokines we observe a marginal (not statistically significant) decrease in concentration when co-stimulated with both TNF-α and IL-6. IL-12p70 appears dependent on the presence of a high concentration of both TNF-α and IL-6, as only the co-stimulated condition showed a significant increase over control. Finally, we observed a strong dependence of IL-8 concentration on high concentrations of TNF-α, as we observe a significant increase in IL-8 concentration in the 100 ng/mL TNF-α condition into the ng/mL range. Additionally, co-stimulation with IL-6 dramatically reduces IL-8 levels as compared with 100 ng/mL TNF-α treatment alone. Finally, there were three cytokines that did not show any changes in concentration (i.e., MCP-1) or were below the lower limit of detection (i.e., IL-2 and TGF-β) in any exposure condition (data not shown).

### 3.6 Changes in gene expression following prolonged exposure to TNF-α

**Figure 7:**
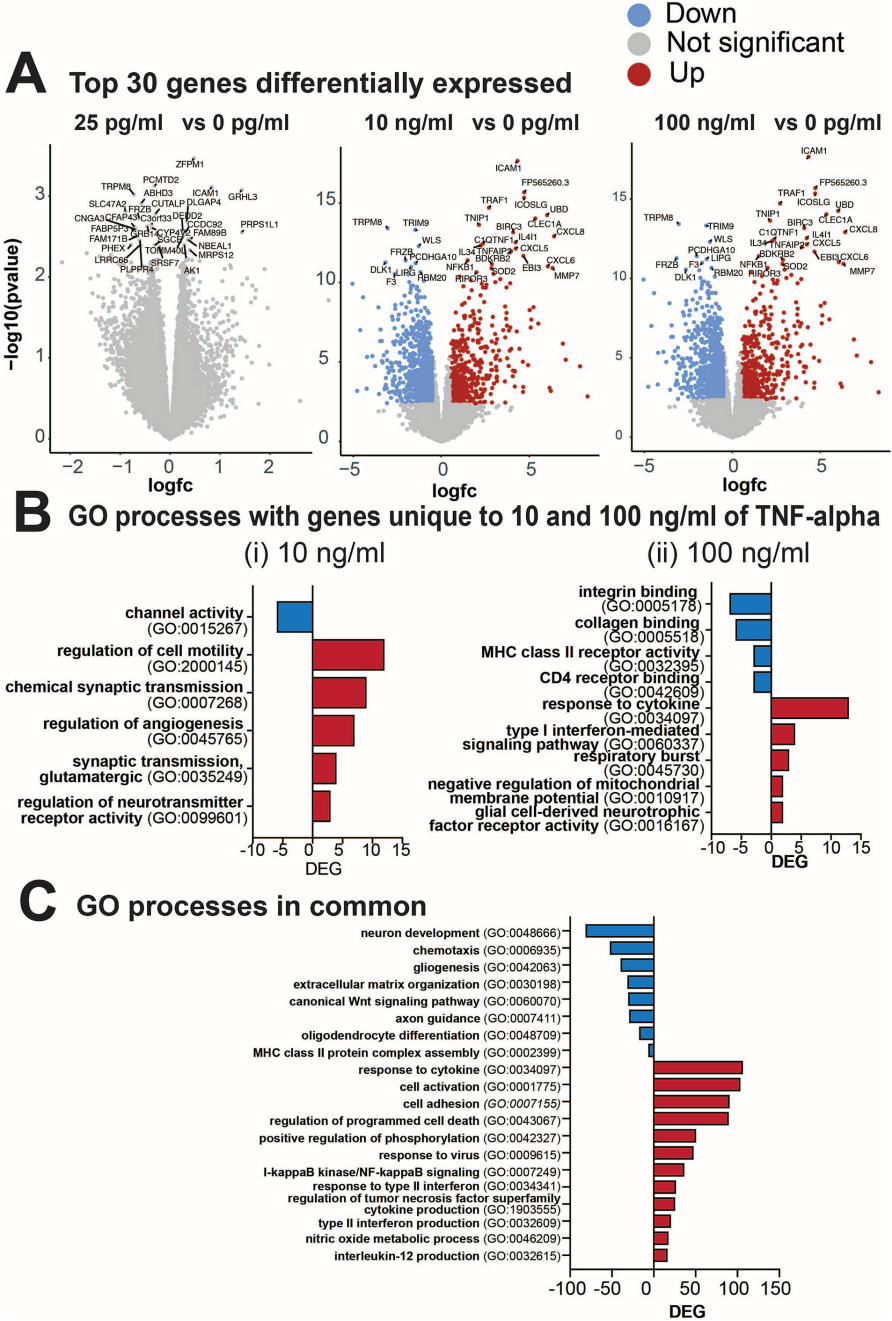
Differential gene expression from cultures exposed to varying concentrations of TNF-α for 7 days. **(A)** Volcano plots of statistical significance vs. magnitude of gene expression between treatment conditions with the top 30 genes highlighted. For the 25 pg/mL condition the top 30 genes highlighted were not significantly differentially expressed, but are still indicated in the plot. **(B)** Gene ontology (GO) categories for genes upregulated (red) or downregulated (blue) that were unique to **(i)** 10 ng/mL TNF-α vs. vehicle and **(ii)** 100 ng/mL TNF-α vs. vehicle. **(C)** Total number of differentially expressed genes (DEG) in each GO category that are commonly upregulated in both 10 ng/mL and 100 ng/mL vs. the control condition.

Next, we performed transcriptomic analysis of cultures treated with various concentrations of TNF-α to gain molecular insights into the strong concentration-dependent effect observed on electrophysiological features and the detected inflammatory cytokines. Differential gene expression (DEG) analysis identified that cultures treated with 10 and 100 ng/mL of TNF-α (but not 25 pg/mL) had altered gene expression profiles at 7 days of exposure (Figure 7A). Specifically, 1,045 genes were differentially expressed in 10 ng/mL TNF-α vs. control and 1,191 genes in 100 ng/mL TNF-α vs. control [DEGs, abs (log_2_FC) > 0.5 and FDR < 0.05; Figure 7A]. As there were no significant alterations in the transcriptome of cultures treated with 25 pg/mL, it suggests that changes in neural functionality were not dependent on transcriptional regulation and expression of genes. For cultures exposed to nanogram concentrations of TNF-α, ontology enrichment analysis of DEGs identified GO biological processes for genes unique to 10 ng/mL TNF-α and were primarily associated with neuronal activity (Figure 7Bi). Specifically, this condition showed a downregulation of genes associated with ion channel activity, and an upregulation of genes involved in synaptic transmission (e.g., chemical synaptic transmission, glutamatergic synaptic transmission, and regulation of neurotransmitter receptor activity). Biological processes for genes unique to 100 ng/mL TNF-α included the downregulation of genes associated with receptor activity (e.g., integrin binding, collagen binding, MHC class II receptor activity, CD4 receptor binding) and the upregulation of genes for processes involved in an immune response (e.g., response to cytokine, type I interferon-mediated signaling pathway, respiratory burst, negative regulation of membrane potential, and glial cell-derived neurotropic factor receptor activity; Figure 7Bii). Biological processes with genes in common for both concentrations of TNF-α (Figure 7C) were associated with an increase in cytokine response or production (e.g., IL-12, type II interferon, and TNF superfamily), and cell death (e.g., regulation of programmed cell death, I-kappaB/NF-kappaB signaling). A decrease in the expression of genes associated with several cellular functions was also detected (e.g., neuron development, gliogenesis, chemotaxis, extracellular matrix organization, axon guidance, oligodendrocyte differentiation).

**Figure 8:**
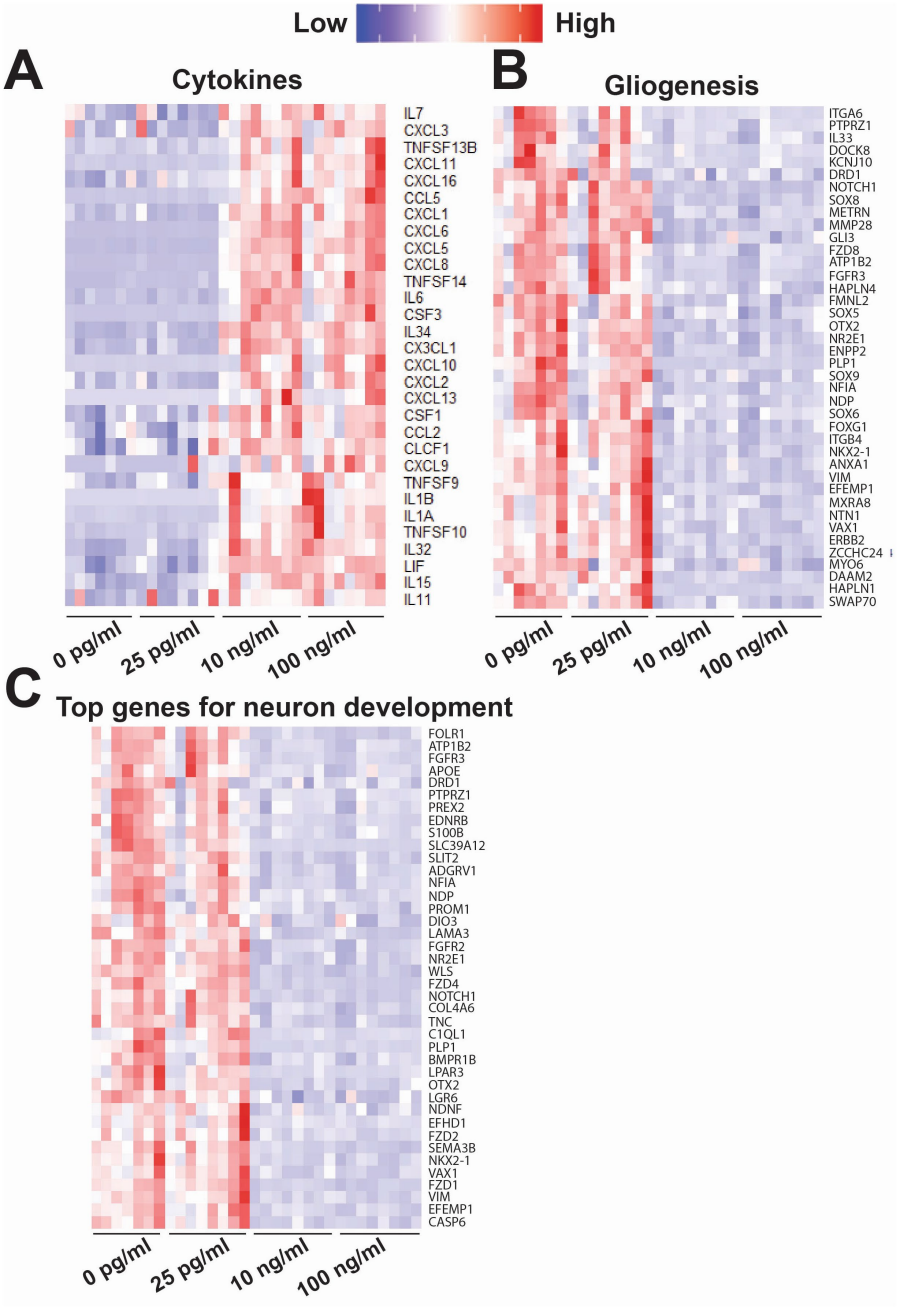
Heatmap of key genes of interest that were differentially expressed following exposure to varying concentrations of TNF-α for 7 days. Each column is a single replicate in the control or TNF-α-treated condition. These genes were sorted into the following biological processes: **(A)** cytokines, **(B)** gliogenesis, and **(C)** neuron development (top 40 genes). Some genes may be included in multiple heat maps if they are involved both biological processes.

In Figure 8, we highlighted genes of interest that were highly upregulated or downregulated in both 10 and 100 ng/mL compared to 0 and 25 pg/mL of TNF-α. For cytokines (Figure 8A) many of the upregulated genes overlapped with the increased cytokine levels observed in the cytokine array (e.g., *IL6, CXCL10, MCP-1/CCL2* and *IL1B*). The upregulation of genes in both the 10 and 100 ng/mL TNF-α treatment condition revealed the increase in specific receptors within the tumor necrosis factor receptor superfamily (e.g., *TNFSF9, TNFSF10, TNFSF13B*, and *TNFSF14*), and chemokines that bind to interleukin-8 (e.g., *CXCL2, CXCL3, CXCL5, CXCL6, CXCL8*, and *CX3CL*) and CXCR3 chemokine (e.g., *CXCL9, CXCL10, CXCL11*, and *CXCL13*) receptors. Genes for growth factor activity were also upregulated (e.g., *IL6, IL7, IL34, IL11, LIF, CSF1, CXCL2*, and *CLCF1*). For gliogenesis (Figure 8B), genes that were downregulated in both the 10 and 100 ng/mL TNF-α suggest a shift in the state of astrocytes, in particular the role of astrocyte differentiation (e.g., *SOX6, SOX8, SOX9, VIM, VAX1, DRD1, FGFR3, PLP1, NOTCH1* and *NR2E1*), and glial cell migration (e.g., *VIM, NTN1, EFEMP1, ATP1B2, DOCK8, PTPRZ1, GLI3, FOXG1*, and *NR2E1*). Additionally, in cultures exposed to nanogram concentrations of TNF-α we observe a decrease in genes involved with neural precursor cell proliferation (e.g., *HAPLN4, SOX5, NFIA, PTPRZ1, GL13, FOXG1, NOTCH1, HAPLN1*, and *NR2E1*), axonogenesis (e.g., *VIM, NTN1, VAX1, ERBB2, FGFR3, NDP, OTX2, METRN, NKX2-1, PTPRZ1, GLI3, GOXG1, NOTCH1*, and *NR2E1*), and genes related to abnormal neuron morphology (e.g., *HAPLN4, CIM, NTN1, VAX1, ERBB2, DRD1, FGFR3, NDP, OTX2, ATP1B2, MYO6, NFIA, PLP1, ENPP2, IL33, GLI3, GOXG1, NOTCH1, KCNJ10*, and *NR2E1*). For neuron development (Figure 8C), the top genes that were downregulated in both the 10 and 100 ng/mL TNF-α conditions were involved in Wnt signaling pathways (e.g., *FZD1, FZD2, FZD4, FGFR2, FGFR3, NDP, LGR6, APOE, WLS, FOLR1, NOTCH1*, and *EDNRB*), regulation of neurogenesis (e.g., F*DZ4, VIM, SLIT2, LPAR3, VAX1, FGFR3, APOE, WLS, PTPRZ1, TNC, NOTCH1, NR2E1, SEMA3B*), and axon guidance (e.g., *LAMA3, COL4A6, SLIT2, VAX1, BMPR1B, NDP, OTX2, LGR6, NKX2-1, TNC, NOTCH1, SMEA3B*).

## 4 Discussion

Pro-inflammatory cytokines, TNF-α and IL-6, have been linked to numerous CNS disorders including neurodegenerative diseases, autism spectrum disorder, psychiatric disorders, and more recently cognitive impairment associated with long-COVID (Lucas et al., 2006; Fernández-Castañeda et al., 2022). However, it is still unclear the extent to which pro-inflammatory cytokines themselves may be responsible for changes in neural function as compared to the underlying neuroinflammatory response. In this study we compared the effect of prolonged exposure of two key cytokines (TNF-α and IL-6) associated with cognitive impairment disorders such as ME/CFS (Islam et al., 2020; Garcia et al., 2014; Mowbray and Yousef, 1991), ‘chemo-fog’ (Murillo et al., 2023; Ganz et al., 2013; Kesler et al., 2013; Pomykala et al., 2013), and long-COVID (Fernández-Castañeda et al., 2022; Schultheiss et al., 2022; Comeau et al., 2023) on neuronal function in a co-culture of human iPSC-derived neurons and primary astrocytes. We selected clinically relevant concentrations of both TNF-α and IL-6 representing ranges found in control or healthy donor patients (Del Valle et al., 2020; Hirzel et al., 2022; Schultheiss et al., 2022; Han et al., 2020) and hospitalized and long-COVID patients (Torabi et al., 2020; Jarius et al., 2022; Ramezani et al., 2022; Schultheiss et al., 2022; Han et al., 2020; Donoso-Navarro et al., 2021; Ulhaq and Soraya, 2020). In addition, we compared changes in cytokine/chemokine secretion and gene expression to gain a better understanding of potential mechanisms underlying the observed functional changes. Notably, it should be recognized that the concentration of cytokines in the serum is not necessarily reflective of the levels within the CSF or CNS microenvironment, especially when considering the close approximation of cytokine producing glial cells and neurons. Therefore, we also tested concentrations that have been previously shown to induce changes in neuron function *in vitro* (Vereyken et al., 2007; Nelson et al., 2002; D’Arcangelo et al., 2000; Kawasaki et al., 2008; Talley et al., 1995; Jayaraman et al., 2021; Hyvärinen et al., 2019), but are significantly higher than detected clinical levels. Experiments using implantable microdialysis catheters have found significantly higher cytokine concentrations within the CNS parenchyma as compared to the CSF, however these results vary and may be dependent on catheter placement, time of sampling, and type of pathology being studied (Zeiler et al., 2017; Woodroofe et al., 1991). Nevertheless, this suggests that the higher TNF-α and IL-6 concentrations taken from previous *in vitro* studies may still hold some physiological relevancy.

Two electrophysiological responses were detected from human iPSC-derived neural networks exposed to TNF-α for 7 days: (1) a concentration-dependent change in overall spiking features; and (2) a concentration-independent effect on burst duration. Cultures exposed to 1 pg/mL and 25 pg/mL TNF-α showed a significant reduction in ISI and a slight (non-significant) increase in the number of spikes and firing rate over time; conversely, cultures exposed to 10 and 100 ng/mL TNF-α show a significant reduction in the number of spikes and firing rate at later timepoints (Figure 1B). This reduction in the number of spikes and spike frequency at high TNF-α concentrations can most likely be attributed to neurotoxic effects. We observed an increase in LDH in the supernatant of cultures exposed to high concentrations of TNF-α for 7 days, which paralleled the reduction in spike frequency (Figure 5A). In addition, time-matched transcriptomic analysis revealed the upregulation of genes involved in GO processes for the regulation of programmed cell death and I-kappaB/NF-kappaB signaling (Figure 7C). While there is still debate on whether TNF-α is neurotoxic on its own (Park and Bowers, 2010; Viviani et al., 2014), there is clear evidence that TNF-α induces neurotoxicity in combination with other pro-inflammatory cytokines or reactive oxygen species (Fischer and Maier, 2015; Liddelow et al., 2017). This suggests that these additional factors maybe naturally present in the cultures or secreted in response to high-concentrations of TNF-α exposure as is the case with pro-inflammatory cytokines such as INF-γ, IL-1β, IL-4, and IL-8 (Figure 6B).

Interestingly, we observed an opposite effect in the response of spiking features to low (picogram) concentrations of TNF-α, with an overall increase in neural activity. We did not observe a change in overall culture health (Figure 2A), inflammatory cytokine secretion profiles (Figure 6), or transcriptome (Figures 7, 8). Cumulative evidence suggests that TNF-α is capable of modulating synaptic strength, either at the pre-synaptic neurotransmitter release probability and/or post-synaptic trafficking of ligand-gated ion channels under homeostatic conditions (Heir and Stellwagen, 2020). Direct TNF-α treatment or its accumulation following activity deprivation induced by tetrodotoxin (TTX) increased the ratio of AMPA to NMDA receptor currents and AMPA receptor surface expression (Stellwagen and Malenka, 2006; Beattie et al., 2002), scaling up the strength of excitatory transmission by increasing the amplitude of miniature excitatory postsynaptic currents (mEPSCs). Similarly, TNF-α was also found to scale down the strength of inhibitory transmission through the reduction in the frequency of miniature inhibitory postsynaptic currents (mIPSCs) (Pribiag and Stellwagen, 2013). In addition, alterations to astrocyte glutamate transport activity may also contribute to the observed increase in neural activity. Astrocytes are known to be an active participant in neural activity and synaptic transmission as part of the “tripartite synapse” (Perea et al., 2009). Rapid changes in neural activity have been previously reported from rat hippocampal-entorhinal complex slice cultures exposed to nanogram concentrations of TNF-α, which was in part attributed to reduced glutamate transport activity in astrocytes (Zou and Crews, 2005). Similarly, Fine et al. (1996) found that fetal human astrocytes exposed to 1 ng/mL TNF-α showed a 30% inhibition in glutamate uptake. In both these cases, the reduction in glutamate transport activity without changes in the protein expression of the glutamate transporters GLAST and GLT-1 (Fine et al., 1996; Zou and Crews, 2005). Finally, it has been shown in both primary mouse cortical and primary rat dorsal root ganglion neurons that treatment with pg/mL concentrations of TNF-α or TNFR-1 and TNFR-2 agonists, respectively, leads to increased voltage-gated sodium channel currents (Chen et al., 2015; Leo et al., 2015), which could also be responsible for the increased neural activity we observe in our study. While these results suggest a non-transcriptomic mechanism underlying the change in neural activity, it is also possible that the transcriptomic change was transient and occurred earlier in the exposure period. Collectively, our findings suggest a concentration-dependent pleiotropic effect of TNF-α on neuronal activity, wherein low levels of the pro-inflammatory cytokine is capable of modulating neural activity in the absence of an inflammatory environment.

An interesting finding in our study was the direct effect of TNF-α, independent of concentration, on the reduction in average burst duration. Bursts are specific spike patterns that are thought to play a critical role in the “neural code” involved in many functions, and include memory, learning, and attention (Lisman, 1997; Friedenberger et al., 2023; Zeldenrust et al., 2018). Many computational models have been developed to gain a better understanding of how different input parameters such as ion channel conductance, synaptic input strengths, and input frequency impact bursting features (Kepecs et al., 2002; Suresh et al., 2016; Shine et al., 2021). TNF-α treatment has been shown to influence all these parameters through direct interactions with ion channels, alterations in surface expression, and changes in gene expression (Vezzani and Viviani, 2015; Viviani et al., 2014). As the changes in burst duration is concentration independent and occurs at low (picogram) levels, it is possible that it is related to the increase in neural activity seen at similar TNF-α concentrations. However, the exact mechanism remains unclear and should be examined in future studies.

Unlike with TNF-α, we did not observe any concentration-dependent effect of prolonged IL-6 exposure on neural activity, but only an overall decrease in the number of spikes in bursts that was present across all IL-6 concentrations tested. This result was somewhat surprising as we found a relatively high concentration (~180 pg/mL) of IL-6 present in the conditioned media from control cultures (Supplementary Figure S6). While this concentration of IL-6 is significantly higher than what is reported in the CSF of control patients, this seemingly higher level of IL-6 is in line with concentrations reported from the CNS parenchyma taken from implanted microdialysis catheters (Woodroofe et al., 1991) and from post-mortem tissue (Li et al., 2009; Vargas et al., 2005). It is important to note that the higher IL-6 concentration found in these studies could be a function of increased IL-6 expression due to neuroinflammatory responses during or prior to sample collection; however other pro-inflammatory cytokines such as TNF-α and IL-1β did not show high levels of expression in postmortem tissue (Li et al., 2009). IL-6 is constitutively expressed within the CNS and plays a significant role in neural stem cell differentiation, synaptic plasticity, memory mechanisms, and neuronal injury repair (Erta et al., 2012; Gruol, 2015). IL-6 primarily acts through the IL-6 receptor system that consists of the signal-transducing glycoprotein 130 (gp130) and the IL-6 receptor. The IL-6 receptor has two forms, a membrane bound form (mIL-6R) or a soluble form (sIL-6R) leading to either the classic or trans-signaling pathways, respectively (Gruol, 2015; Rose-John et al., 2023). Additionally, there is another signaling pathway, cluster signaling, in which the IL-6/mIL-6R complex forms on one cell and then activates membrane-bound gp130 on a neighboring cell (Rose-John et al., 2023). It is hypothesized that these different signaling modalities may lead to different responses to IL-6, with classic signaling typically associated with homeostatic processes, while trans-signaling is involved with neuroinflammatory pathways (Hunter and Jones, 2017). This difference in signaling modalities may be responsible to the significant reduction in the number of spikes in bursts we observe, even in cultures exposed to low concentrations of IL-6. Even at low concentrations, the added IL-6 may alter the balance between classical vs. trans-signaling and thereby alter neuronal or network functions. Additionally, both gp130 and mIL-6R have been shown to be localized to both pre- and postsynaptic membranes (D’Arcangelo et al., 2000), which also suggests that the addition of “free” IL-6 may be influencing neuronal function.

Compared to TNF-α, the response of neural activity to IL-6 treatment is less clear in literature. While IL-6 has a clear role in learning and memory, exposure to IL-6 has been shown to produce both excitatory and inhibitory effects depending on the neuron type, concentration, timeframe, and method of exposure (Gruol, 2015; Vezzani and Viviani, 2015; Viviani et al., 2014). For example, transgenic mice overexpressing IL-6 in astrocytes (GFAP-IL6) show an increase in excitatory activity from EEG recordings, while slice cultures from the same GFAP-IL-6 mice show reduced long-term potentiation (LTP) and lower spontaneous firing rates (Campbell et al., 1993; Bellinger et al., 1995; Nelson et al., 1999). Similarly, Balschun et al. (2004) demonstrated that IL-6 expression is increased in both hippocampal slice cultures and in the hippocampus of freely moving rats in response to LTP, and blocking IL-6 via a neutralizing antibody increased LTP maintenance. Cultured Purkinje neurons displayed both increased and reduced activity in response to IL-6, with cultures exposed to 10 ng/mL IL-6 showing reduced activity, while cultures exposed to 1 ng/mL showing increased activity (Nelson et al., 1999). These changes were attributed to increases in the input resistance of the neurons and increased response to AMPA receptor activation. Conversely, Garcia-Oscos et al. (2012) found IL-6 exposure reduced the amplitude of evoked inhibitory postsynaptic currents (eIPSCs), potentially through the reduction of GABA_A_ receptor density at the synapse, with no change to evoked excitatory postsynaptic currents (eEPSCs). Previous studies have demonstrated that reduced input resistance is characteristic of strongly burst firing neurons (Williams and Stuart, 1999), suggesting that an increase in input resistance may be responsible for the decrease in the number of spikes in bursts. However, in our study we did not observe a concentration dependent effect on the number of spikes in bursts, suggesting that other additional mechanisms are likely at play.

Finally, we investigated the effects of co-stimulation of both TNF-α and IL-6 on neural and network functionality. We compared the effect of co-stimulation with two concentrations of TNF-α (25 pg/mL and 100 ng/mL) to capture both the concentration-dependent increase or decrease in spiking activity, along with 10 ng/mL IL-6, as IL-6 did not show any concentration-dependent effects on neural functionality. We found that cultures treated with TNF-α showed significant levels of IL-6 within the conditioned media, while IL-6 did not induce TNF-α secretion by the cultures (Supplementary Figure S6). Interestingly, the addition of 10 ng/mL IL-6 appeared to counteract some of the cytotoxic effects of high levels of TNF-α, as cultures treated with both 10 ng/mL IL-6 and 100 ng/mL TNF-α no longer show a significant increase in extracellular LDH as compared to controls (Figure 5C). This is perhaps not surprising as IL-6 has been shown to play a neuroprotective role during many pathological conditions of the CNS (Kummer et al., 2021). Cytokine data supports this potential neuroprotective role of IL-6 within our study, as treatment with IL-6 led to increased concentrations of IL-4 and IL-10 within the conditioned media (Figure 6A). Both IL-4 and IL-10 are considered anti-inflammatory cytokines and have also been shown to be neuroprotective during pathological conditions within the CNS (Porro et al., 2020; Wang et al., 2024; Spittau, 2017). Despite the reduction in neurotoxicity, cultures co-stimulated with both 100 ng/mL TNF-α and 10 ng/mL IL-6 still showed reduced neural activity and burst duration similar to cultures exposed to 100 ng/mL TNF-α alone. Conversely, when cultures were co-stimulated with 25 pg/mL TNF-α and 10 ng/mL IL-6, there were no significant changes in any electrophysiological features as compared to vehicle. Interestingly, co-stimulation with either concentration of TNF-α led to the loss of changes to the number of spikes in bursts seen in IL-6 treated cultures. These results suggest that at low concentrations of TNF-α, TNF-α and IL-6 may have antagonistic effects on neural function, however at high concentrations, electrophysiological changes associated with TNF-α exposure become dominant.

While we observe clear changes in neural function as a direct result of prolonged TNF-α and IL-6 exposures in human-relevant co-cultures of neurons and astrocytes, the limitation of this model is that it is without microglia, the innate immune cell of the brain. It is important to remember that cognitive impairment associated with long-COVID and other post-viral infection disorders is most likely caused by a multitude of factors, most notably the sustained innate and adaptive immune responses that persist beyond viral clearance. Nevertheless, these results indicate that the increased concentrations of key inflammatory cytokines associated with long-COVID can impact neural function irrespective of other immune responses and may be a component of cognitive impairment. The results from this study may also provide insights into other conditions of the CNS which are associated with chronic neuroinflammation and increased TNF-α and IL-6 concentrations including neurodegenerative diseases (Zhang et al., 2023) and neuropsychiatric conditions (Hong et al., 2016). Surprisingly, these changes to neural function were not accompanied by significant changes to neuronal synchrony (Figures 2, 3B, 4B). Further studies examining changes at the level of network structures and communities (Cadena et al., 2020) would be beneficial to elucidate effects of prolonged cytokine exposure on neural network function.

## Data Availability

The datasets presented in this study can be found in online repositories. The names of the repository/repositories and accession number(s) can be found at: https://www.ncbi.nlm.nih.gov/geo/, GSE279308.

## Footnotes

^1^ https://legendplex.qognit.com

^2^ https://www.bioinformatics.babraham.ac.uk/projects/fastqc/

^3^ https://usegalaxy.eu/
