# Supplementary material for: Direct effects of prolonged TNF-α and IL-6 exposure on neural activity in human iPSC-derived neuron-astrocyte co-cultures
Source: Front Cell Neurosci. 2025 Feb 12;19:1512591. doi: 10.3389/fncel.2025.1512591 (PMC11860967; doi:10.3389/fncel.2025.1512591)
Supplement: Supplementary file 1 [file Table_1.docx]

Supplementary Material


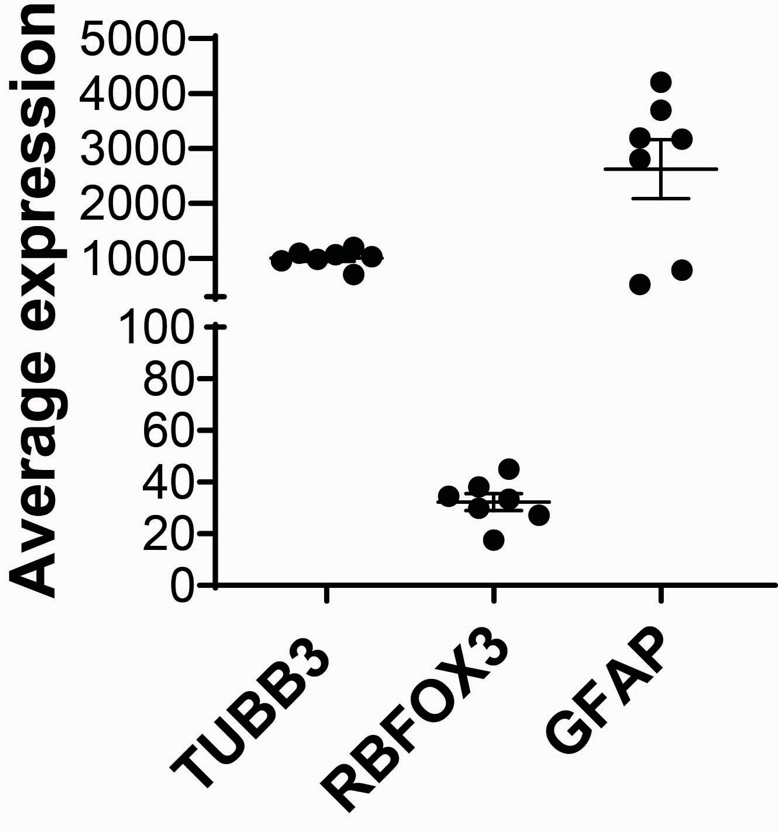


**Supplementary Figure 1. Expression levels of cell-type specific mRNA markers.** The average mRNA expression level is shown for the neuronal markers, tubulin beta 3 (TUBB3) and RNA binding fox-1 homolog 3 (RBFOX3), and the astrocyte marker, glial fibrillary acidic protein **(**GFAP), from the co-culture system at ~32 DIV which was treated with vehicle. Data is expressed as mean ± SEM.

**
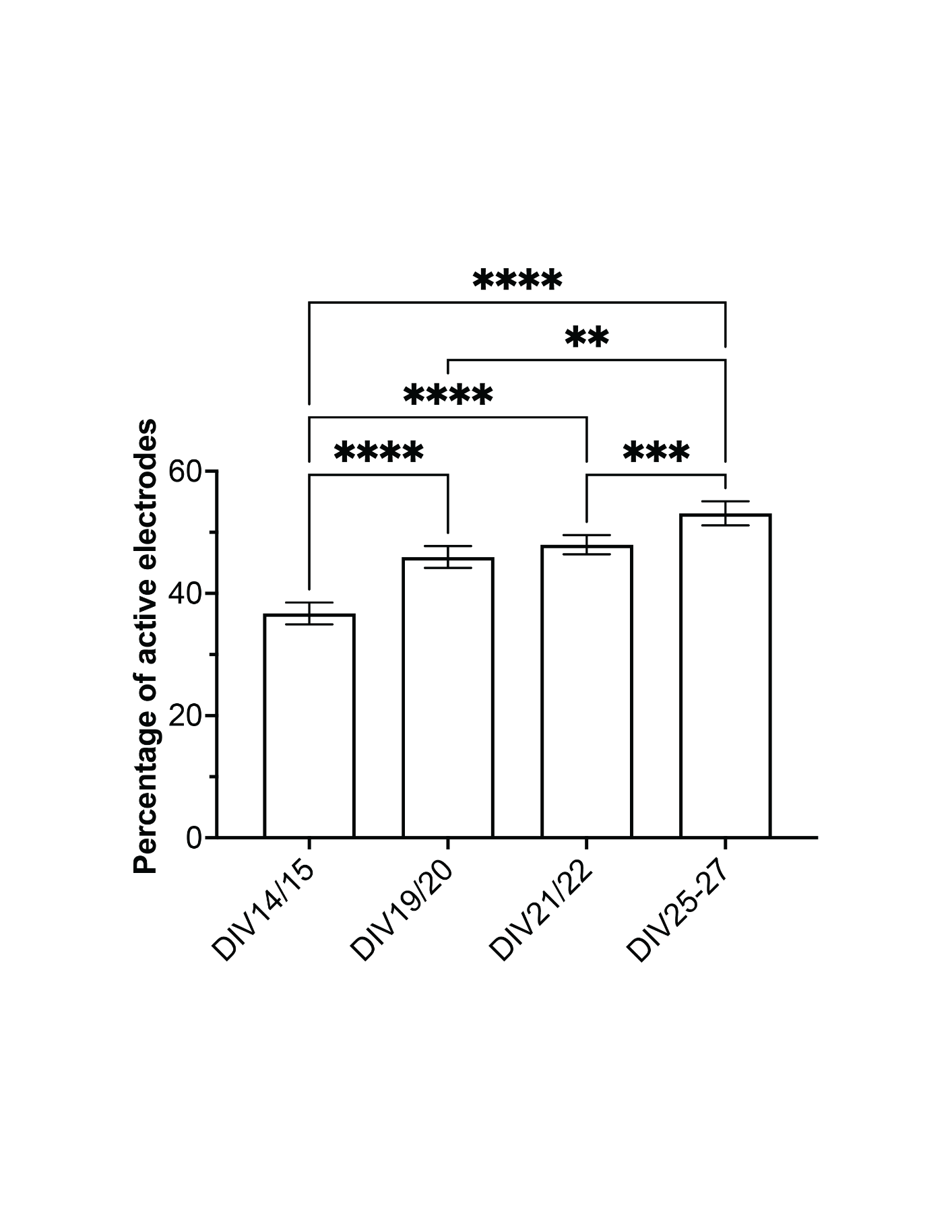
**

**Supplementary Figure 2.** Percentage of electrodes that detected an action potential spike in the cultures during development. The percentage of active electrodes increases over time and appears to stabilize around 25 days in vitro (n = 83-114 from 5 independent seedings). Data is shown as mean ± SEM; ***p*<0.01, ****p*<0.001 and **** *p*<0.0001 (as determined by a repeated measures two-way ANOVA followed by Tukey’s post hoc test to compare time points).

**
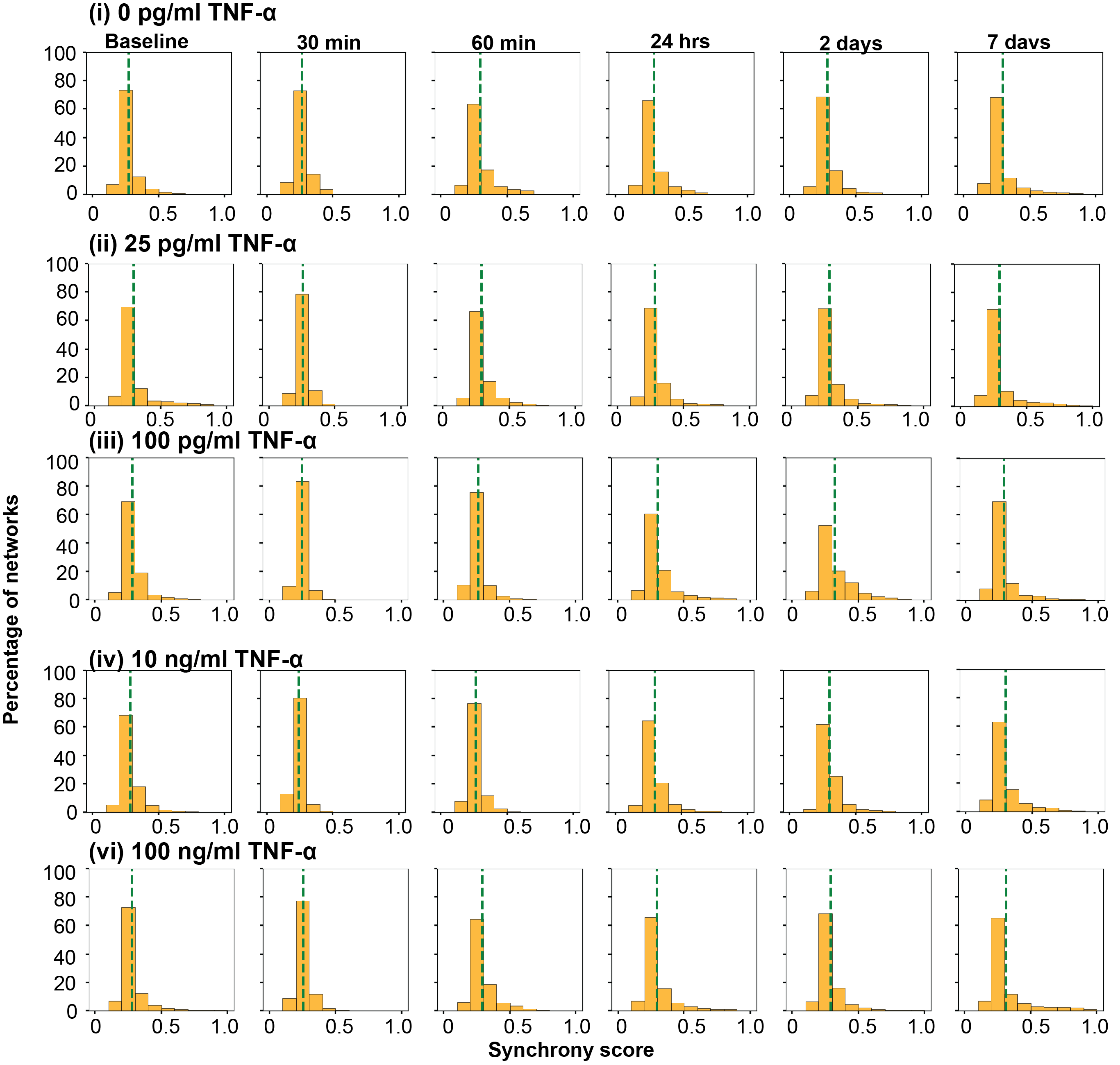
**

**Supplementary Figure 3.** Histograms summarizing the distribution of synchrony scores across all electrode parings across all TNF-α treatments. The green line indicates the average synchrony score from all electrode pairs.


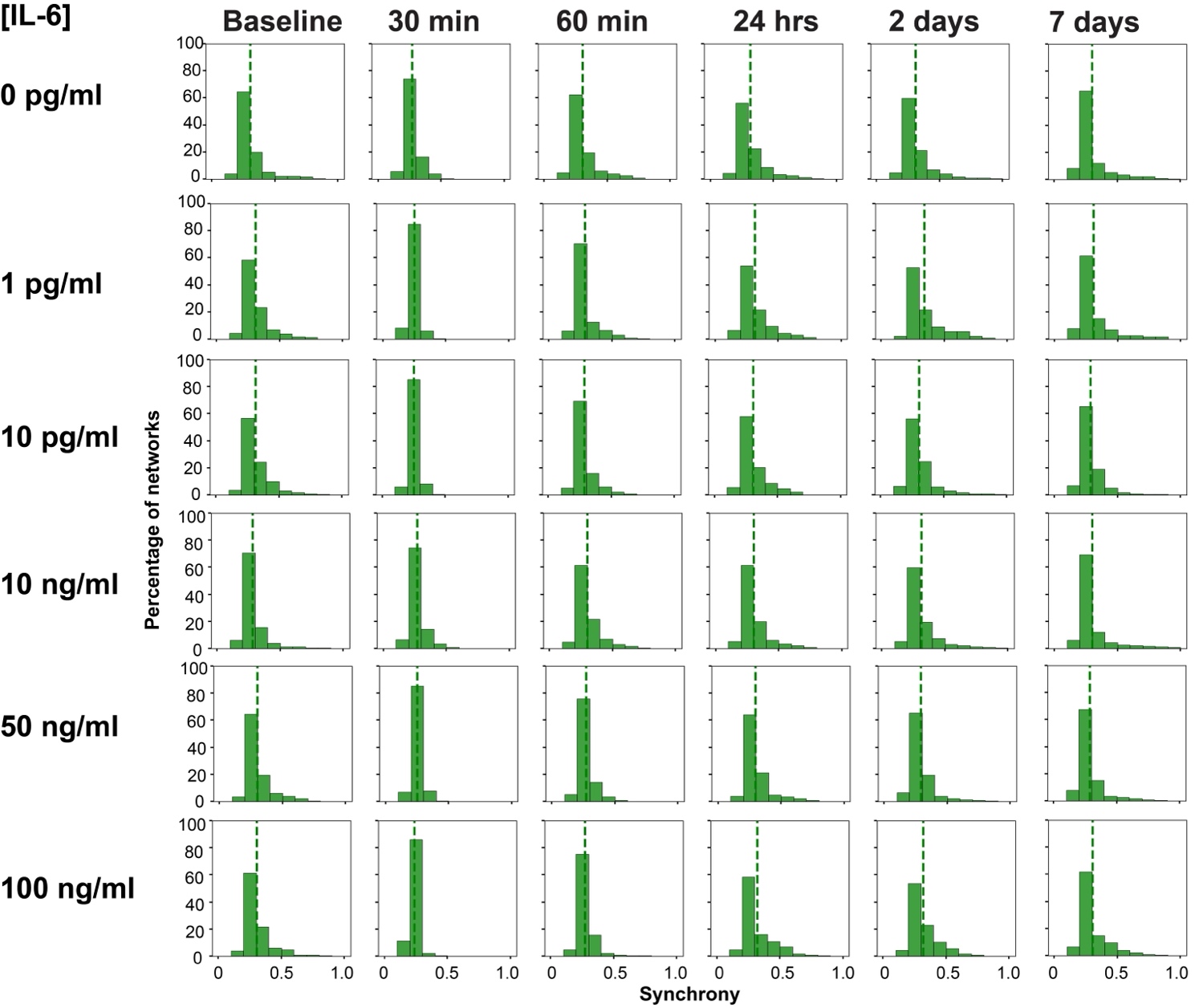


**Supplementary Figure 4:** Histograms summarizing the distribution of synchrony scores across all electrode parings across all IL-6 treatments. The green line indicates the average synchrony score from all electrode pairs.


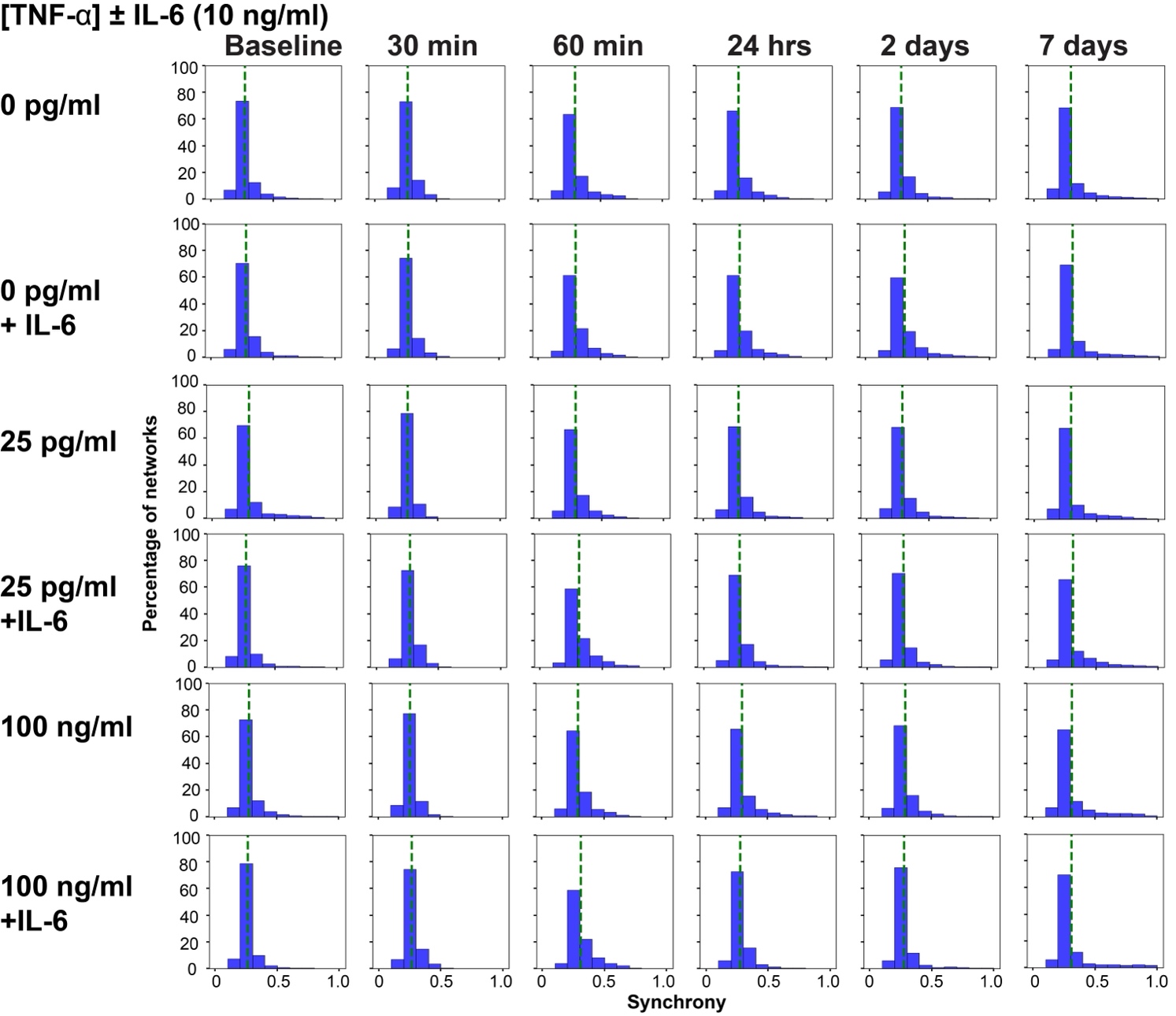


**Supplementary Figure 5:** Histograms summarizing the distribution of synchrony scores across all electrode parings across all TNF-α and IL-6 exposure combinations. The green line indicates the average synchrony score from all electrode pairs.


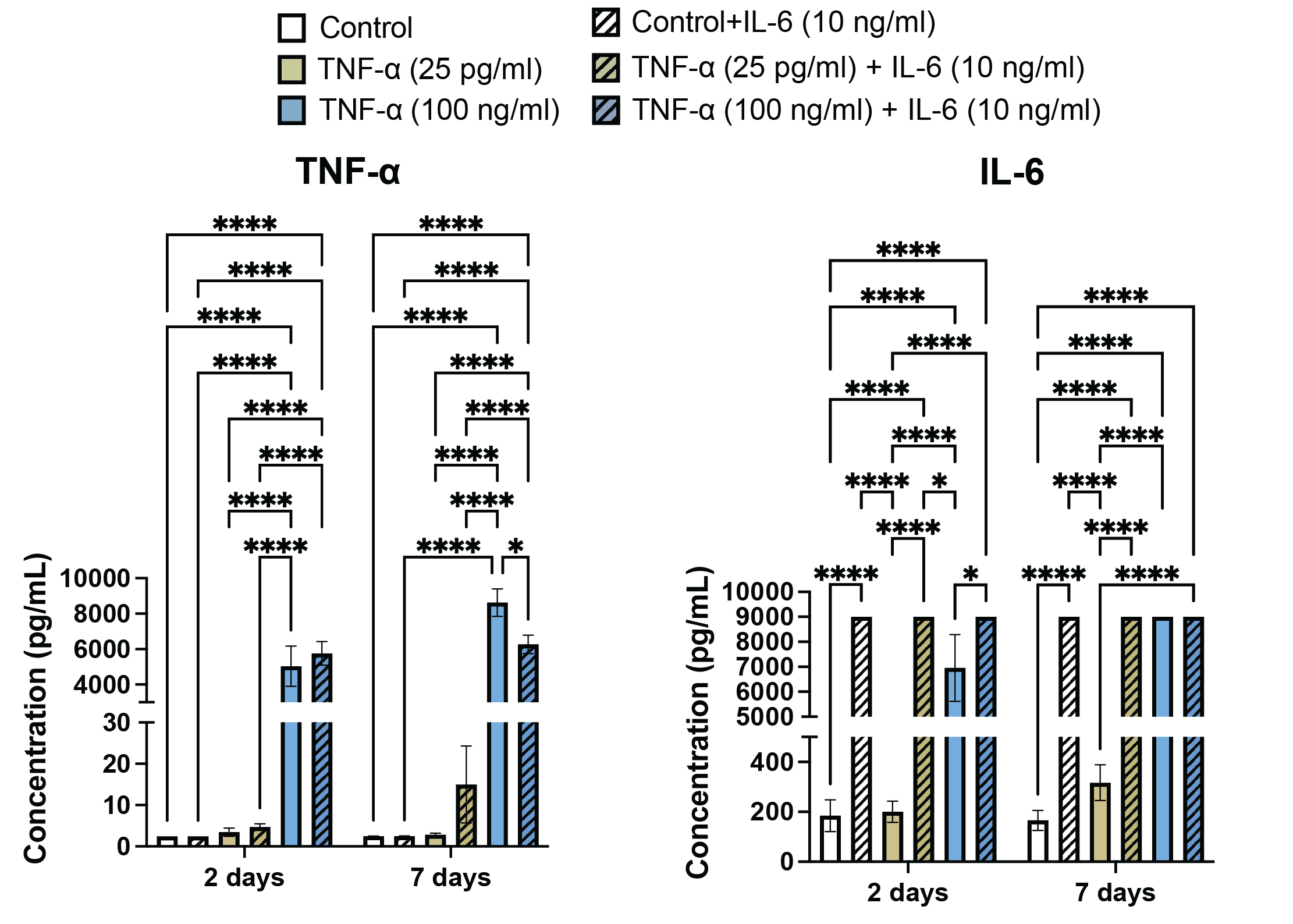


**Supplementary Figure 6:** Concentrations of TNF-α and IL-6 from conditioned media of co-cultures exposed to both TNF-α and IL-6 (n = 4-12 from at least 2 independent seedings). Data is shown as mean ± SEM; **p*<0.05, ***p*<0.01, ****p*<0.001, and *****p*<0.0001 (as determined by a one-way ANOVA for each timepoint followed by a post hoc Tukey’s test). Some significant post hoc comparisons, in which the treatment conditions have no overlap (i.e. 25 pg/mL TNF-α + 10 ng/mL IL-6 vs 100 ng/mL TNF-α) are not shown to improve clarity.
